# Risk of Major Adverse Cardiovascular Events in Patients With Rheumatoid Arthritis

**DOI:** 10.7759/cureus.12246

**Published:** 2020-12-23

**Authors:** Kheraj Mal, Ratan Kumar, Farah Mansoor, Navneet Kaur, Anil Kumar, Sidra Memon, Amber Rizwan

**Affiliations:** 1 Cardiology, National Institute of Cardiovascular Diseases, Sukkur, PAK; 2 Cardiology, Khairpur Medical College, Nawabshah, PAK; 3 Internal Medicine, Jinnah Sindh Medical University, Karachi, PAK; 4 Internal Medicine, Adesh Institute of Medical Sciences and Research, Buchu Kalan, IND; 5 Family Medicine, Jinnah Post Graduate Medical Center, Karachi, PAK

**Keywords:** rheumatoid arthritis, myocardial infarction, cardiovascular events, autoimmune diseases

## Abstract

Introduction

Rheumatoid arthritis is a chronic, inflammatory, and multisystem disease, which, along with the joints, can involve the cardiovascular system. The treatment of rheumatoid arthritis or rheumatoid arthritis itself can lead to atherosclerosis, which is considered one of the major causes by which it can affect the cardiovascular system. In this study, we will assess the risk of cardiovascular events in patients with rheumatoid arthritis as compared to the general population.

Method

This case-control study was conducted from January 2018 to November 2018. Two-hundred twenty-two (222) patients with diagnosed rheumatoid arthritis were included as cases in the study. Two-hundred eleven (211) patients were included in the study as the control group (patients without rheumatoid arthritis). All the data were recorded in a self-structured questionnaire.

Result

Participants with rheumatoid arthritis also showed an increased risk of myocardial infarction (MI) by an odds ratio of 2.50 (95% CI; 0.77-8.14). There was also an increased risk of cardiovascular death in participants with rheumatoid arthritis by an odds ratio of 1.99 (0.58-6.71).

Conclusion

The study suggests that rheumatoid arthritis along with joint inflammation can also affect the cardiovascular system. Hence, a multidisciplinary team of rheumatologists and cardiologists should manage patients suffering from rheumatoid arthritis, which will improve morbidity and mortality in such patients.

## Introduction

Rheumatoid arthritis (RA) is an autoimmune disorder affecting multiple parts of the body. It is more common in females than in males [1]. This disease can involve multiple systems other than joints due to systemic inflammation, including the cardiovascular system [2]. RA speeds up atherosclerosis in the vessels, leading to an increased risk of myocardial infarction (MI) and stroke. These can cause high mortality among patients suffering from RA [3]. The overall incidence of cardiovascular events among RA patients is relatively more common as compared to the general public, thus making RA an independent risk factor for cardiovascular diseases like diabetes mellitus [4-6].

Various risk factors and mechanisms are identified to explain the increased risk of cardiovascular events in patients with rheumatoid arthritis. These mechanisms include undertreatment of cardiovascular risk factors in RA patients, adverse events associated with the treatment of RA, and the inflammatory process in RA, which leads to accelerated atherosclerosis [7].

Currently, to the best of our knowledge, there is no study based in Pakistan assessing cardiovascular risk in patients with rheumatoid arthritis. More insight and local data are required to assist clinicians in discussing the management of cardiovascular risk in patients with RA.

## Materials and methods

This case-control study was conducted in a tertiary-care hospital in Pakistan. Two-hundred twenty-two (222) patients with a confirmed diagnosis of rheumatoid arthritis were included in the study from January 2018 to November 2018, with the help of rheumatologists. Two-hundred eleven (211) participants, matched for age and gender with the case group, without a known diagnosis of rheumatoid arthritis, were included from the outpatient department of cardiology unit as the control group.

All participants' age, body mass index, hypertension status, smoking status, gender, and physical activity were noted in a self-structured questionnaire. Physical inactivity included patient responses 'no exercise' or 'one to two times a month' versus 'regular exercise one or more times a week'. Their blood was drawn and sent to the laboratory for cholesterol levels.

Patients were followed for 12 months or for the development of a major cardiovascular event (MACE), whichever came first. In this study, MACE was defined as patients having a nonfatal stroke, nonfatal myocardial infarction, and cardiovascular death. Two-hundred ten (210) participants from the case group completed the study while two-hundred five (205) participants from the control group completed the study. Only participants who completed the study were included in the final analysis.

Statistical analysis was done using the Statistical Package for the Social Sciences (SPSS) v. 23.0 (IBM Corp., Armonk, NY). Continuous variables were analyzed via descriptive statistics and were presented as mean data while standard deviations (SDs) were presented as categorical data. The t-test and chi-square were applied as appropriate. The odds ratio was calculated to measure the association between RA and cardiovascular events. A p-value of less than 0.05 meant that the difference between the groups is significant and the null hypothesis is void.

## Results

The characteristics and risk factor profile were similar between both groups, except that physical inactivity was significantly more common in participants with rheumatoid arthritis (p-value < 0.00001) (Table 1).

**Table 1:** Comparison of participants with rheumatoid arthritis vs. participants without rheumatoid arthritis n: number; SD: standard deviation; kg: kilogram; mg: milligram; dL: decilitre; NS: non-significant

Characteristics	Participants with Rheumatoid Arthritis (n=210)	Participants without Rheumatoid Arthritis (n=205)	p-value
Age in years (Mean ± SD)	46 ± 12	48 ± 13	NS
Male %	42	49	NS
BMI greater than 25 kg/m^2^ (%)	33	31	NS
Cholesterol level greater than 200 mg/dL	32	35	NS
Hypertensive (%)	32	35	NS
Current smokers (%)	17	19	NS
Physically inactive (%)	76	48	< 0.00001

Participants with rheumatoid arthritis had an increased risk of myocardial infarction by an odds ratio of 2.50 (95% CI; 0.77-8.14). There was also an increased risk of cardiovascular death in participants with rheumatoid arthritis by an odds ratio of 1.99 (0.58-6.71) (Table 2).

**Table 2:** MACE in participants with rheumatoid arthritis vs. participants without rheumatoid arthritis n: number; OR: odds ratio; CI: confidence interval; NS: non-significant; MACE: major cardiovascular event

Major Adverse Cardiovascular Event (MACE)	Participants with Rheumatoid Arthritis (n=210)	Participants without Rheumatoid Arthritis (n=205)	OR (CI, 95%)	p-value
Non-Fatal Stroke	3 (1.43%)	3 (1.47%)	0.97 (0.19-4.89)	NS
Non-Fatal Myocardial Infarction	11 (5.23%)	4 (1.95%)	2.50 (0.77-8.14)	NS
Cardiovascular Death	8 (3.80%)	4 (1.95%)	1.99 (0.58-6.71)	NS

## Discussion

In the present study, we included 210 patients with RA and 205 patients without RA that visited the cardiology department, analyzed whether they developed a MACE, and calculated the odds ratio. It was found that patients with RA had an increased risk of MI by an odds ratio (OR) of 2.50 (95% CI; 0.77-8.14). Similar results were also found in another study, which included 4,363 RA patients from 15 countries and demonstrated a 9.3% prevalence rate of cardiovascular (CV) morbidity among patients and an overall 3.2% prevalence for MI. They also observed that patients with RA were 1.9% more prevalent to develop stroke, however, our results were not significant for the development of stroke [8]. Interestingly, Lindhardsen et al., in their study, observed that the 70% increase risk of MI in RA was identical to the risk of MI in patients without RA who were 10 years older, with male patients having a more uniform graph. This finding points to the role of age and gender in the development of MI in patients with RA [7]. Furthermore, patients with RA are related to a 60% higher risk of death due to cardiovascular diseases (CVD) in comparison to the general population [9]. This is in correspondence to our finding of an increase in mortality due to CVD in RA patients by OR of 1.99.

Multiple studies have found a link between RA and CVD by considering RA as a systemic state of chronic inflammation [10]. Although, the mechanism is poorly understood. In one study, Karpouzas and colleagues observed the formation of coronary plaques in RA patients and compared the results with matched controls and discovered non-calcified plaques were found in arteries of 54% of patients with asymptomatic RA patients and only 21% in the control group. They also found the extent and severity of these plaques were more common in patients suffering from RA [11]. The pathophysiology behind this is explained by Charles-Schoeman C in his research, which demonstrated the decreased function of high-density lipoprotein (HDL) in RA patients with severe disease activity using a 28 joint count (Disease Activity Score 28 > 5.1) [12]. Normally, HDL functions as an antiatherogenic by its ability to promote cholesterol efflux from arterial cells and reduce low-density lipoprotein (LDL) oxidation, both of which are necessary pathways in atheroma formation [13-14]. Therefore, the withdrawal of the protective effect of HDL on arteries albeit the normal HDL level could be one of the pathways of increased risk of CVD in patients with RA [12]. Another inciting event in atherosclerotic plaque formation is endothelial dysfunction [15]. Recently, Sidibe et al. found high levels of the extracellular domain of vascular endothelial cadherin (VE-90) in the plasma serum of patients with RA. VE-cadherin is an endothelium adhesion molecule and is a major molecule for endothelium cohesion. These circulating high levels manifest endothelium dysfunction [16]. Moreover, antibodies of RA such as anti-cyclic citrullinated peptide (anti-CCP) antibodies and rheumatoid factor immunoglobulin M (RF IgM) as well as inflammatory markers C-reactive protein (CRP) and erythrocyte sedimentation rate (ESR) have been positively associated with impaired endothelial function and progressive carotid plaque, respectively. High levels of these markers in RA patients might predict the risk and severity of CVD [12,17-18]. However further studies are required in exploring the direct pathway linking CVD and RA markers.

It is a well-known fact that physical inactivity has a deteriorating effect on a patient’s general health, causing a wide variety of diseases such as CVD [19]. Patients of RA lead a sedentary lifestyle due to joint inflammation and pain [20]. The present study found that 74% of patients with RA were physically inactive (p=< 0.00001) than patients without RA. Lack of physical activity in such patients can accelerate the atherosclerotic process and causes endothelial impairment. A decrease in resistance exercise has been shown to elevate systemic inflammation, all of which can increase the risk of development of MI [20]. Weight loss, control of blood sugar levels, and maintenance of blood pressure are all associated with increased physical activity [21].

Lastly, a frequently used anti-inflammatory medication in RA, prednisone has shown long-term adverse effects, such as hypertension, weight gain, and impaired glucose tolerance, which are all modifiable risk factors for MI [22-23]. Other pharmacotherapy for RA includes tumor necrosis factor (TNF) alpha inhibitors, methotrexate, and hydroxychloroquine, which have been shown to reduce the likelihood of MI [13,24].

MI is one of the major causes of premature death in patients suffering from RA. Some studies have found an equal risk of increase of MI in patients with RA in comparison with diabetic patients [25]. Lowering systemic inflammation, assessing RA markers as an early sign of CVD, prescribing cardioprotective medication for RA treatment, and encouraging patients to increase physical activity will prove to be beneficial in reducing the risk and mortality rate due to MI in patients with RA. It is, therefore, essential to consider RA as an independent risk factor for CVD.

## Conclusions

Rheumatoid arthritis is a chronic debilitating condition affecting a variety of organ systems, especially affecting the blood vessels, leading to increased mortality due to myocardial infarction. Hence, to prevent such grave complications, rheumatologists and cardiologists should work together to screen the patients already suffering from rheumatoid arthritis for a possibility of impending myocardial infarction. Diagnostic and management plans should be devised to control such outcomes, which will help in improving the morbidity and mortality of these patients.

## References

[1] Scott DL, Wolfe F, Huizinga TW (2010). Rheumatoid arthritis. Lancet.

[2] Sandoo A, Zanten J, Metsios GS, Carroll D, Kitas GD (2011). Vascular function and morphology in rheumatoid arthritis: a systematic review. Rheumatology.

[3] Kaplan MJ (2006). Cardiovascular disease in rheumatoid arthritis. Curr Opin Rheumatol.

[4] Bacon PA, Stevens RJ, Carruthers DM, Young SP, Kitas GD (2002). Accelerated atherogenesis in autoimmune rheumatic diseases. Autoimmun Rev.

[5] del Rincón ID, Williams K, Stern MP, Freeman GL, Escalante A (2001). High incidence of cardiovascular events in a rheumatoid arthritis cohort not explained by traditional cardiac risk factors. Arthritis Rheum.

[6] Solomon DH, Goodson NJ, Katz JN (2006). Patterns of cardiovascular risk in rheumatoid arthritis. Ann Rheum Dis.

[7] Lindhardsen J, Ahlehoff O, Gislason GH, Madsen OR, Olesen JB, Torp-Pedersen C, Hansen PR (2011). The risk of myocardial infarction in rheumatoid arthritis and diabetes mellitus: a Danish nationwide cohort study. Ann Rheum Dis.

[8] Naranjo A, Sokka T, Descalzo MA (2008). Cardiovascular disease in patients with rheumatoid arthritis: results from the QUEST-RA study. Arthritis Res Ther.

[9] Meune C, Touzé E, Trinquart L, Allanore Y (2009). Trends in cardiovascular mortality in patients with rheumatoid arthritis over 50 years: a systematic review and meta-analysis of cohort studies. Rheumatology (Oxford).

[10] Smolen JS, Aletaha D, McInnes IB (2016). Rheumatoid arthritis. Lancet.

[11] Karpouzas G, Ahmadi N, Choi T (2010). Lower prevalence and severity of ‘vulnerable’ coronary plaque in a-TNF-exposed asymptomatic patients with rheumatoid arthritis. Arthritis Rheum.

[12] Charles-Schoeman C, Lee YY, Grijalva V (2012). Cholesterol efflux by high density lipoproteins is impaired in patients with active rheumatoid arthritis. Ann Rheum Dis.

[13] Charles-Schoeman C (2012). Cardiovascular disease and rheumatoid arthritis: an update. Curr Rheumatol Rep.

[14] Schaftenaar F, Frodermann V, Kuiper J, Lutgens E (2016). Atherosclerosis: the interplay between lipids and immune cells. Curr Opin Lipidol.

[15] Incalza MA, D'Oria R, Natalicchio A, Laviola L, Giorgino F (2018). Oxidative stress and reactive oxygen species in endothelial dysfunction associated with cardiovascular and metabolic diseases. Vascul Pharmacol.

[16] Sidibé A, Mannic T, Arboleas M (2012). Soluble VE-cadherin in rheumatoid arthritis patients correlates with disease activity: evidence for tumor necrosis factor α-induced VE-cadherin cleavage. Arthritis Rheum.

[17] Hjeltnes G, Hollan I, Førre Ø, Wiik A, Mikkelsen K, Agewall A (2011). Anti-CCP and RF IgM: predictors of impaired endothelial function in rheumatoid arthritis patients. Scand J Rheumatol.

[18] Giles JT, Post WS, Blumenthal RS (2011). Longitudinal predictors of progression of carotid atherosclerosis in rheumatoid arthritis. Arthritis Rheum.

[19] Ricci NA, Cunha AIL (2020). Physical exercise for frailty and cardiovascular diseases. Adv Exp Med Biol.

[20] Verhoeven F, Tordi N, Prati C, Mougin F, Wendling D (2016). Physical activity in patients with rheumatoid arthritis. Joint Bone Spine.

[21] Myers J, Kokkinos P, Nyelin E (2019). Physical activity, cardiorespiratory fitness, and the metabolic syndrome. Nutrients.

[22] Krasselt M, Baerwald C (2016). Efficacy and safety of modified-release prednisone in patients with rheumatoid arthritis. Drug Des Devel Ther.

[23] Boateng S, Sanborn T (2013). Acute myocardial infarction. Dis Mon.

[24] Rempenault C, Combe B, Barnetche T, Gaujoux-Viala C, Lukas C, Morel J, Hua C (2018). Metabolic and cardiovascular benefits of hydroxychloroquine in patients with rheumatoid arthritis: a systematic review and meta-analysis. Ann Rheum Dis.

[25] Ráček V, Němec P (2012). Rheumatoid arthritis - an independent risk factor for cardiovascular disease [Article in Czech]. Vnitr Lek.

